# The traditional use of wild edible plants in pastoral and agro-pastoral communities of Mieso District, eastern Ethiopia

**DOI:** 10.1186/s41182-023-00505-z

**Published:** 2023-02-23

**Authors:** Muhidin Tahir, Abdulaziz Abrahim, Tigist Beyene, Gedefa Dinsa, Tilahun Guluma, Yosef Alemneh, Patrick Van Damme, Umer Seid Geletu, Amin Mohammed

**Affiliations:** 1 Department of Biology, College of Natural and Computational Sciences, Oda Bultum University, P.O. Box 226, Chiro, Ethiopia; 2 Department of Chemistry, College of Natural and Computational Sciences, Oda Bultum University, P.O. Box 226, Chiro, Ethiopia; 3 Department of Food Sciences and Post Harvesting Managements, College of Agriculture, Oda Bultum University, P.O. Box 226, Chiro, Ethiopia; 4 Laboratory for Tropical and Subtropical Agriculture and Ethnobotany, Department of Plants and Crops, Faculty of Bio-Science Engineering, Ghent University, Coupure Links 653, 9000 Ghent, Belgium; 5 Faculty of Tropical AgriSciences, Czech University of Life Sciences Prague, Kamycka 129, 165 21 Prague 6-Suchdol, Czech Republic; 6 Department of Animal Science, College of Agriculture, Oda Bultum University, P.O. Box 226, Chiro, Ethiopia; 7 Department of Plant Sciences, College of Agriculture, Oda Bultum University, P.O. Box 226, Chiro, Ethiopia

**Keywords:** Ethnobotany, Indigenous knowledge, Wild edible plants, Use values, Mieso District, Ethiopia

## Abstract

**Background:**

The populations in Ethiopia have developed their indigenous knowledge to use, manage and conserve wild edible plants (WEPs). In the eastern part of Ethiopia, wild edible plants are used as a means of survival during times of food shortage and as dietary supplements. Documenting the traditional and cultural use of wild food plants is a vital step in obtaining baseline data for investigating nutritional values and possible side effects, preserving indigenous knowledge, and ultimately interesting in wild edible plant conservation. However, their significance, management and utilization have not been documented in Mieso District. Therefore, this study aimed to provide documentation of wild edible plant use associated with ethnobotanical knowledge in Mieso District, eastern Ethiopia.

**Methods:**

An ethnobotanical study of wild edible plant species was conducted from March 2021 to May 2022. A total of 120 (72 males and 48 females) informants were selected using the snowball method. Data collection methods, including semistructured interviews, direct observation and field walks were used. Data were analysed using descriptive statistics, including independent sample *t* test and analysis of variance (ANOVA). We calculated use values (UVs) to analyse the relative cultural importance of each plant species.

**Results:**

A total of 41 wild edible plant species belonging to 33 genera in 21 families were documented to be used as food sources both during times of food shortage and as dietary supplements. Family Malvaceae was best-represented with 6 species, followed by Fabaceae and Rhamnaceae (4 species each). The dominant growth form (habit) was shrubs (30 species), followed by trees (11 species). The most widely used plant parts were fruits (covering 39 species, 95%). The largest number (23 species) was collected from forest habitats only, followed by both village and forest habitats (8 species). The majority of wild edible plants (28 species or 68%) were consumed only during famine or in the time of food shortage followed by supplementing staple foods (9 species or 22%). Wild edible plants in Mieso are used for multiple other uses, including for fodder, fuel, medicine, construction, cosmetics and bee keeping. Twenty-three species were mentioned for fodder use, followed by fuel purpose (21 species) and medicinal value (13 species). The species that had the highest use values were *Flacourtia indica* (Burm.f.) Merr. (1.4), *Carissa spinarum* L. (1.1), *Ziziphus spina-christi* (L.) Desf. (0.6), *Grewia villosa* Willd. (0.5), *Cordia monoica* Roxb. (0.3) and *Opuntia ficus-indica* (L.) Mill. (L.) (0.2). Most WEPs were collected from March to May (*Badheysa*) (33 species). The highest mentioned wild edible plant sold in the market was *F. indica* (Burm.f.) Merr. mentioned by 20 informants, followed by *Z. spina-christi* (L.) Desf. (14).

**Conclusion:**

The people in Mieso use wild plants as supplementary food to cultivated crops, during famine, and many could be utilized for day-to-day human consumption. Some plants in the district provide cash income for local people. However, deforestation (54%), drought (22%) and agricultural expansion (12%) were the highest threats to wild plants in Mieso District. Hence, on-site and off-site conservation would help protect wild plant resources in Mieso, eastern Ethiopia.

**Supplementary Information:**

The online version contains supplementary material available at 10.1186/s41182-023-00505-z.

## Background

Wild edible plant use and collection are parts of identity for the local people and cultural history of different ethnic groups, traditions and pride [1]. The consumption of these plant resources is essential for the livelihood strategies of many people across the world [2–6]. In addition, it is important for supplementing daily dietary or as famine foods during scarcity in developing populations [7–9]. They play an important role in eliminating poverty and providing a source of income in developing populations [10]. They can also serve to provide important genetic resources for obtaining new crops that have better yields and tastes [11, 12]. Moreover, WEP resources have been established as an important element in ecosystem-based adaptations and coping strategies to lessen food scarcity globally [13, 14]. In addition, WEPs are also important in retaining the significance of cultures to rural populations in developing nations [15–17].

Nearly 300,000–500,000 flora species are described to be present on the Earth’s planet, of which 30,000 are edible, and nearly 7000 species are wild plants collected for food [6]. However, the wide range of crop cultivations in conjunction with the revolution of industries and changes in lifestyles have resulted in the underutilization of wild edible plant resources [18]. In Ethiopia, the presence of different topographies associated with various edaphic conditions, diverse ethnic groups and various food cultures yields diverse indigenous knowledge and flora resources [19]. Furthermore, Ethiopia is also one of the eight centres of crop product diversity worldwide [20]. Nearly 6500–7000 flora species—among which 12% endemic plant species were estimated to be present in the country [21]. Nearly 413 wild/semiwild edible flora species in Ethiopia have been documented, and most wild edible fruits in the country are used by humans [22]. Most rural populations in developing countries, including Ethiopia, are unable to obtain sufficient foods via conventional means [23–25]. Hence, they rely on wild plant resources to supplement their diet, particularly during periods of food shortage or famine [26], and their consumption is more common in areas, where food insecurity is prevalent [27].

Approximately 81 million people and 85% of ethnic groups in Ethiopia are residents of rural areas; thus, they traditionally rely on wild plant resources for various purposes, including for food, medicine, cash income, fodder and construction [4, 25]. Despite the great role of wild plants in Ethiopia, little has been done to properly document and investigate wild edible plant resources and related knowledge [22]. In addition, wild edible plant use and knowledge in Ethiopia is being threatened, because it mostly known with people who are older and disappearing, and the knowledge is transferred orally [22]. The rural people in the Oromia region in general [4, 28] and the people of Mieso District in particular highly depend on wild edible plant resources. However, wild edible plants in Mieso District are threatened by deforestation, agricultural expansion, and the indigenous knowledge of WEP is also disappearing due to oral-based transmission.

Cultural domains in different communities are the key starting points for investigating the perceptions of local people, and they are also important aspects for understanding cultural settings [22, 27]. Free listing [29] is an important method to understand elements of the cultural domain, and several researchers have used this method [30–32]. Any local specific knowledge, which is retained in the community, documented and transferred, has uses that are vital for subsistence, and it relies on social transmissions to family members or within the community [33, 34]. Innovation is the initial step of knowledge acquisition [35], whereas observation, familiarizing natural resources and providing help to adults are the first steps of knowledge transmission associated with natural resources and their uses [36]. Traditional knowledge on wild food plant uses has been kept in the memory of ethnic groups as a heritage and passed orally through generations [37, 38]. Nonetheless, the traditional culture of natural resources is deteriorating, leading to the loss of indigenous knowledge [39]. Moreover, wild edible plant knowledge and uses are decreasing due to the development of agricultural and modern food industries in conjunction with negative perception of WEPs, consumption times associated with the collection of WEPs, and lack of interest or reluctance of younger generations to try, use and even get to know WEPs [40, 41]. Hence, documenting local knowledge is important before disappearing along with people who have upheld it [42]. The following research questions are sought to be answered: (1) What are the wild food species used in the area? (2) How available are WEPs throughout the year? (3) Which parts of the plants are used? (4) Do they have other uses, i.e., for fodder, fuel, medicine, construction, cosmetics and bee keeping? (5) How is the knowledge of WEPs transmitted to the family and/or within the community? (6) What are the main threats to WEPs and uses? (7) Which wild edible plants are sold in the market? Besides, to date, there has been no documentation on wild edible plant use associated with indigenous knowledge in Mieso District.

Therefore, this study aimed to provide documentation of the indigenous knowledge associated with wild edible plant uses in Mieso District, eastern Ethiopia.

## Methods

### Description of the study area

**Fig. 1:**
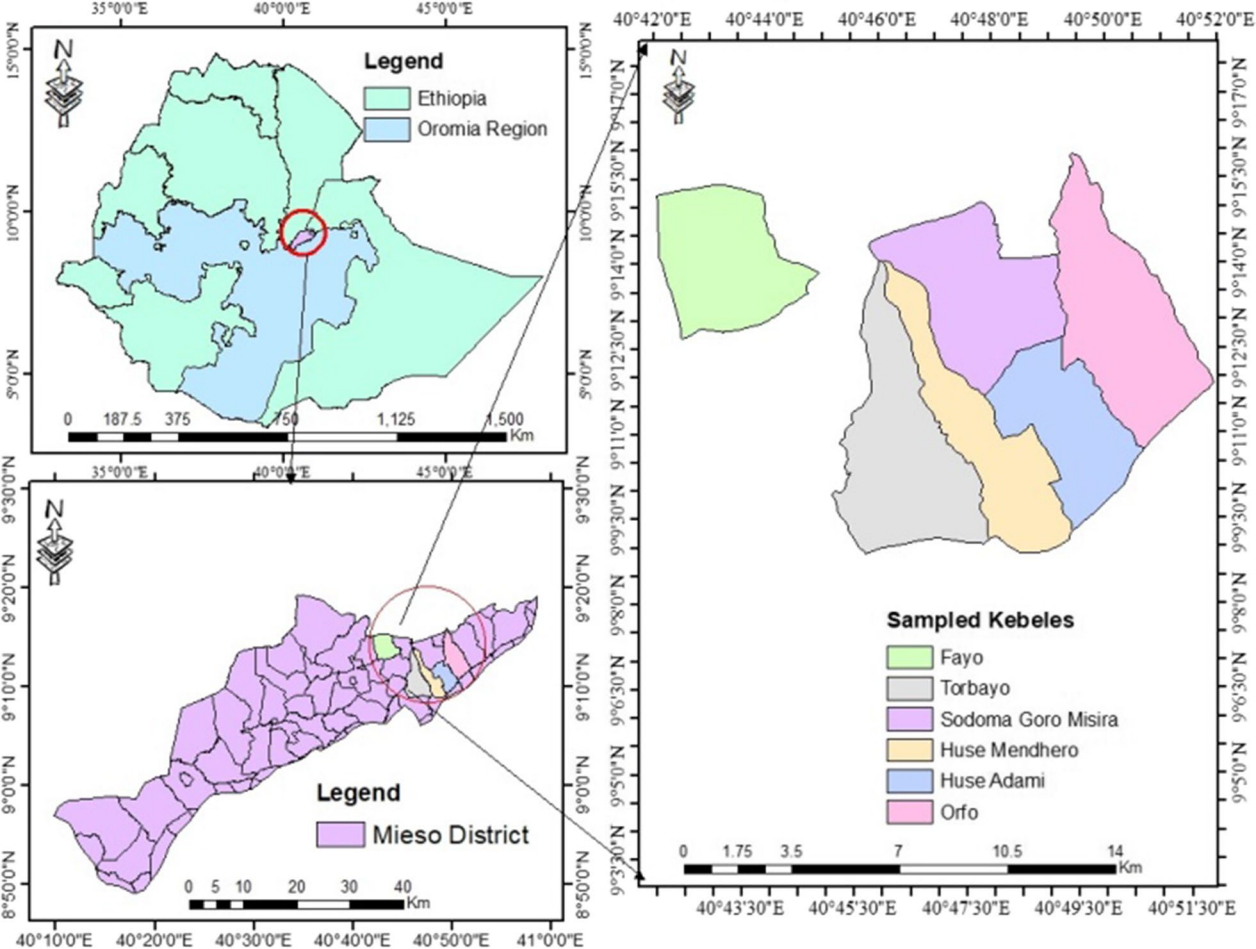
Map of the study area

The study was conducted in Mieso District located at 40° 9″ 30′ E and 40° 56″ 44′ E and 8° 48″ 12′ N and 9° 19″ 52′ N, with elevations ranging from 900 to 1600 m above sea level in the Oromia Regional State of Ethiopia (Fig. 1). It is bounded by Guba Koricha to the south, Afar regional state to the west, Somali regional state to the north, Doba to the east and Chiro District to the southwest. The district is situated 300 km southeast of Addis Ababa (capital city of Ethiopia). The mean annual temperature of Mieso District is 21 °C, while the average annual rainfall is 790 mm. Drought is a major problem, and as a result, crops fail in most years due to the lack of even distribution of rainfall (Mieso District Agricultural office, unpublished data of 2015).

The total population of the Mieso District was at 202,580, of whom 115,836 were men and 86,744 women, 40,777 were urban dwellers, and 161,803 were rural inhabitants. A total of 34,405 male-led and 9563 female-led households were counted in the district. The four main ethnic groups in Mieso are Oromo (88.09%), Somali (5.77%), Amhara (3.46%) and Argoba (0.66%); all other ethnic groups made up 2.02% of the total population [43]. Most of the population (88.37%) speaks *Afan Oromo*, followed by Amharic (5.61%) and Somali (5.29%) [43]. The district is agroecologically classified as a semiarid lowland. Pastoralists make up 80% of the total population, agro-pastoralists 15%, and 5% are engaged in crop-livestock production. Crops such as sorghum, maize, sesame, and haricot beans are commonly cultivated in the district. Out of a total land area of 196,026 ha, 11.5% is arable, 10.7% is crop land, 23.7% is cultivable if water is available, 9% is grazing land, 8.9% is pasture, and 28.7% is forest. The common livestock populations are cattle (156,331), goats (173,927), camels (60,294), sheep (47,705), donkeys (43,197) and chickens (55,612). The major feeding resource for livestock comes from rangelands, which cover approximately 73,658 ha (38%) of the total land area (Mieso District Agricultural office, unpublished data of 2015).

### Data collection

Ethnobotanical data collection was carried out from March 2021 to May 2022 during the three seasons in the study area. A total of six study *kebeles* (wards; smallest administrative units), i.e., Torbayo (9° 14″ 19′N and 40° 61″ 1′E), Sodoma Goro Misira (9° 13″ 41′N and 40° 47″ 30′E), Huse Mendhero (9° 11″ 42'N and 40° 49″ 11′E), Huse Adami (9° 11″ 42′N and 40° 49″ 12′E)*,* Orfo (9° 14″ 21′N and 40° 46″ 15′E) and Fayo (9° 14″ 16′N and 40° 43″ 45′E), were selected purposively from a total of 31 *kebeles* in the study district based on the recommendation of the local elder, local authority and presence of wild edible plants. A total of 120 informants (72 males and 48 females) were selected by the snowball method. Data were collected using semistructured interviews [29] involving local name of the plants, availability, used plant parts, habitats, times of consumption, condition of used plants (fresh/dried) and threats to WEPs knowledge and use, and current conservation status and other uses following Pei and Long [44].

Direct observations and field walks were also carried out to obtain information on wild edible plant use. In addition, the socioeconomic characteristics of informants (ages, gender, education levels and occupations) were also surveyed. Focus group discussions with knowledgeable respondents were carried out to confirm/validate information focusing on acceptance of wild edible plants, adverse effects, threats to wild edible plants and conservation status. We also conducted market surveys in the Mieso market near the village to record information on marketable wild edible plants in the daily life of local peoples. Consent for data collection was obtained from the head of each village and individual informants before conducting the ethnobotanical survey, and field study and interviews were conducted following the ethical guidelines of the International Ethnobiology Society (International Society of Ethnobiology. ISE Code of Ethics. 2008).

Identification of plant species was made in the field and national herbarium, using different taxonomic literature, by comparison with voucher specimen references, and using various flora books of Ethiopia and Eritrea [45–52]. Plant specimens involving local name, date of collection, collection number, collectors’ name, description and location of the plant were pressed, labelled and taken to the National Herbarium, Addis Ababa for further identifications and deposited in Oda Bultum University. The International Code of Botanical Nomenclature (ICBN) naming system was followed for species identification and naming.

### Data analysis

The general WEP use values were calculated using all uses mentioned by informants for each cited species using the formula adapted from Phillips and Gentry [53]; it was done considering an interview of a single informant following Bortolotto et al. [54]:

$${\text{UVs }} = \Sigma U{\text{s}}/n,$$

where UVs is the use value of a particular species “s”, *n* refers to the total number of informants involved in the study (*n* = 120), and Us is the number of mentions of use cited by each respondent for a particular species “s.”

In addition, the influence of sociodemographic factors (gender, age, education level and occupation of the respondents) on wild edible plant knowledge was analysed using one-way analysis of variance (one-way ANOVA) using SPSS (version 20). Furthermore, we used Microsoft spreadsheet (Excel) to draw and tabulate graphs and compute sums and percentages.

## Results and discussion

### Sociodemographic characteristics of respondents

Different sociodemographic factors of informants, i.e., education levels, males/females, age groups, and occupations were included in the study. Most of the informants were males (72), whereas 48 were females. Most informants’ ages ranged from 21 to 40 years (80), whereas 27 were between 41 and 60 years and 13 were above 61. The majority of the informants (53) were illiterate, followed by elementary school 49 (grades 1–4) and high school and diploma (18) (Table 1). The main occupation of the respondent is agriculture (65), followed by homemakers (37) and students (18).

**Table 1:** Wild edible plant knowledge among different social groups (*n* = 120)

Parameter	Category	*N*	Mean	DF	Sum of Square	Mean square	*F* value	*p* value
Gender	Males	72	4.3	1	10.841	10.841	2.21	0.062
	Females	48	3.5	1				
Age groups (in years)	21–31	47	2	5	429.325	86	122.5	0.0001*
	32–41	34	4					
	42–51	18	7					
	52–61	9	9					
	62–71	7	9					
	> 72	5	9.5					
Education level	Illiterate	53	5.5	2	175.8	88	113.8	0.0001*
	Elementary school	49	2.4					
	High school and diploma	18	1					
Occupation	Farmers	65	6.1	2	111.6	55.8	30	0.0001
	Students	18	3.6					
	Homemakers	37	3.4					

*Shows a significant difference at *p* < 0.05 between averages of the paired categories

Significant differences in knowledge of wild edible plants between different social groups were observed. Comparison of wild edible plant knowledge between male and female informants using a two-tailed independent sample *t* test showed that there was an insignificant knowledge difference (*p* > 0.05) between them (Table 1). This shows that family members share their knowledge equally. This finding is in line with previous ethnobotanical studies [55, 56]. However, some studies have shown that females often tend to have better traditional knowledge, because they mostly participate in activities that support their households and sustenance to their family [57–60]. This culturally acquired knowledge is combined with day-to-day information to improve their subsistence for their families [61].

There was also a significant difference in wild edible plant knowledge between informants’ age groups (*p* < 0.05); older people knew more WEPs than adults and young people (Table 1), as was also indicated in other similar ethnobotanical studies [6, 54]. This could be explained by the reluctance or lack of interest of the younger generation to gain/transfer knowledge of wild edible plants, as also observed in an ethnobotanical study from Argentine Patagonia [62].

From an education status perspective, a significant WEP knowledge difference (*p* < 0.05) between different education levels was observed; this shows that more knowledge was held by the illiterates and respondents with lower formal education than by more learned/educated informants (Table 1). This might be due to illiterates and respondents with lower formal education relying on agricultural activity, whereas nonagricultural jobs are preferred by more educated people [6].

From the perspective of informants’ occupations, the knowledge difference in the mean number of wild edible plants reported between different occupations was significant (*p* < 0.05). Farmers hold more knowledge than informants with other occupations (Table 1). Economic development, improvement of living conditions, and preference for nonagricultural activities by most people could explain the loss of WEPs knowledge transmission and retention [6]. This result is in agreement with the study by Cheng et al. [6] in the sense that farmer informants had more knowledge of WEPs than informants involved in other occupations (Table 1).

### Diversity of wild edible plants, their growth forms and use values

Overall, 41 WEPs belonging to 33 genera in 21 families were documented to be used by the people of Mieso District both during the times of food shortage and as dietary supplements (Table 2). The number of wild edible plant species documented in Mieso District was higher than that from similar ethnobotanical studies in Ethiopia, i.e., in Berek Natural Forest, (*n* = 34) were mentioned [4]; in Kefira market, (*n* = 22) [63]; in Chilga District, (*n* = 33) [64]; in Quara District, (*n* = 36) [65]; in Kara and Kwego, (*n* = 38) [66]; and in Yilmana Densa and Quarit Districts, (*n* = 32) [67].

**Table 2:** List of wild edible plants used by Mieso people

Scientific name	Local name	Family	Habit/Habitat	Plant part used	Collection month	Other use	Use value	Citation	Collection number
Abutilon mauritianum (Jacq.) Medik.	Daneysa	Malvaceae	Shrub/Forest and Village	Fruit	Dec, Jan, Feb	Fodder	0.013	–	MT 010
Acacia prasinata Asfaw	Dodoti	Fabaceae	Shrub/Village	Fruit	Mar, Apr, May	Construction, Fuel, fodder	0.08	[28]	MT 018
Acacia senegal (L.) Willd.	Sobesa	Fabaceae	Shrub/Forest	Bark	Jun, Jul, Aug	Fuel, fodder	0.03	[28, 68, 69]	MT 027
Acacia tortilis (Forssk.) Hayne	Tedeche	Fabaceae	Tree/Forest	Fruit	Mar, Apr, May	Fodder	0.013	[28]	MT 032
Acokanthera schimperi (A.DC.) Schweinf.	Keraro	Apocynaceae	Shrub/Forest	Fruit	Mar, Apr, May	Construction, fuel, fodder	0.08	[68]	MT 007
Balanites aegyptiaca (L.) Delile	Bedeno	Zygophyllaceae	Tree/Forest	Fruit	Mar, Apr, May	Construction, fuel, fodder, medicine for human (bloating)	0.13	[27, 28, 64, 65, 68, 70, 77]	MT 037
Berchemia discolor (klotzsch) Hemsl.	Jejeba	Rhamnaceae	Tree/Forest	Fruit	Mar, Apr, May, Sep, Oct, Nov	Fuel, fodder	0.2	[28, 68]	MT 001
Breonadia salicina (Vahl) Hepper & J.R.I.Wood	Dabessa	Rubiaceae	Tree/Forest	Fruit	Sep, Oct, Nov	Medicine (against cancer), fuel, construction, fodder	0.1	–	MT 040
Carissa spinarum L.	Agamsa	Apocynaceae	Shrub/Forest	Fruit	Mar, Apr, May, Sep, Oct, Nov	Fuel, construction, fodder, bee keeping, hair cosmetics	1.14	[4, 27, 28, 64, 67-69, 77, 82, 83]	MT 002
Commiphora africana (A.Rich.) Endl.	Hammessa	Burseraceae	Tree/Forest	Fruit	Mar, Apr, May	Fodder	0.1	[28]	MT 023
Cordia africana Lam.	Wodeysa	Boraginaceae	Tree/Village	Fruit	Mar, Apr, May, Sep, Oct, Nov	Construction, fuel	0.04	[4, 27, 28, 64, 65, 67-69, 70, 77]	MT 030
Cordia monoica Roxb.	Mendhero	Boraginaceae	Shrub/Swamp area	Fruit	Mar, Apr, May, Sep, Oct, Nov	Medicine (Leaves; itching), Construction, fuel, fodder	0.34	[28, 68]	MT 006
Dombeya aethiopica Gilli	Danisa	Malvaceae	Shrub/Forest	Fruit	Mar, Apr, May	Fodder	0.04	–	MT 034
Dovyalis abyssinica (A.Rich.) Warb.	Shimbr-qoli	Salicaceae	Shrub/Swamp area	Fruit	Mar, Apr, May	Shade	0.01	[4, 27, 64, 67, 69]	MT 011
Embelia schimperi Vatke	Hanqu	Primulaceae	Shrub/Forest and Village	Fruit	Mar, Apr, May	Fodder	0.013	[67, 69]	MT 041
Euclea racemosa L.	Mi'essa	Ebenaceae	Shrub/Forest and Village	Fruit	Sep, Oct, Nov	Medicine (roots is crushed and applied on the infected eyes of livestock and for diarrhoea), Fuel, construction, fodder	0.013	[28]	MT 012
Flacourtia indica (Burm.f.) Merr.	Hudha	Salicaceae	Shrub/Forest	Fruit	Jun, Jul, Aug	Medicine (cancer), fuel, construction, fodder	1.4	[27, 82]	MT 013
Grewia rothii DC.	Haroreysa	Malvaceae	Shrub/Forest	Fruit	Mar, Apr, May	Fodder, shade	0.06	–	MT 024
Grewia villosa Willd.	Ogomdii	Malvaceae	Shrub/Forest	Fruit	Sep, Oct, Nov	Soap (hair), cosmetic, construction, fuel, fodder	0.5	[66, 68]	MT 005
Grewia ferruginea Hochst. ex A.Rich.	Tatessa	Malvaceae	Shrub/Forest	Fruit	Sep, Oct, Nov	Medicine (cancer), fuel, construction, fodder and fence	0.4	[4, 68-70]	MT 014
Hibiscus micranthus L.f.	Qunce	Malvaceae	Shrub/Forest	Fruit	Jun, Jul, Aug	Fodder, tooth brush	0.3	–	MT 025
Hydnora abyssinica A.Br.	Tuqa	Hydnoraceae	Shrub/Forest	Root	Jun, Jul, Aug	Medicine (cancer)	0.1	–	MT 029
Mimusops kummel Bruce ex A.DC.	Oladi	Sapotaceae	Shrub/Forest	Fruit	Mar, Apr, May, Sep, Oct, Nov	Construction, fuel	0.05	[64, 67, 68]	MT 035
Morella salicifolia (Hochst ex.A.Rich) Verdc. & Polhill	Bika	Myricaceae	Shrub/Forest	Fruit	Mar, Apr, May	Fuel	0.04	[28]	MT 015
Myrsine africana L.	Kechu	Primulaceae	Shrub/Forest	Fruit	Mar, Apr, May	Medicine (Intestinal parasites)	0.04	[27, 69]	MT 028
Maytenus undata (Thunb.) Blakelock	Fanta fullassa	Celastraceae	Shrub/Forest	Fruit	Mar, Apr, May, Sep, Oct, Nov	Fuel, fodder, fence	0.03	–	MT 009
Opuntia ficus-indica (L.) Mill.	Tini	Cactaceae	Shrub/Forest and village	Fruit	Mar, Apr, May	Medicine (anemia), Fuel, fodder, fertilizer,	0.2	[4, 27, 28, 67, 68]	MT 016
Opuntia humifusa (Raf.) Raf.	Hadami	Cactaceae	Shrub/Forest and village	Fruit	Sep, Oct, Nov	Fuel	0.2	Not reported	MT 017
Pappea capensis Eckl. & Zeyh.	Biqa	Sapindaceae	Tree/Forest	Fruit	Mar, Apr, May	Fodder	0.05	[27]	MT 021
Plectranthus montanus Benth.	Berbarisha	Lamiaceae	Shrub/Forest and village	Fruit	Jun, Jul, Aug, Sep, Oct, Nov	Fence	0.03	Not reported	MT 026
Prunus africana (Hook.f.) Kalkman	Koki	Rosaceae	Tree/Village	Fruit	Mar, Apr, May	Fodder	0.03	[4]	MT 033
Psidium guajava L.	Zeituna	Myrtaceae	Shrub/Village	Fruit	Mar, Apr, May	Fodder	0.04	Not reported	MT 038
Rosa abyssinica R.Br.	Gora	Rosaceae	Shrub/Forest	Fruit	Mar, Apr, May, Sep, Oct, Nov	Fuel, fence, fodder	0.05	[64, 67, 69]	MT 019
Rhus natalensis Bernh. ex C.Krauss	Debobesa	Anacardiaceae	Shrub/Forest	Fruit	Sep, Oct, Nov	Medicine (cancer), fuel, construction, fodder	0.1	[4, 28, 64, 68, 83]	MT 039
Solanum americanum Mill.	Mujulo	Solanaceae	Shrub/Forest and village	Fruit	Jun, Jul, Aug	Fodder	0.01	[68]	MT 036
Syzygium guineense (Willd.) DC.	Bedessa	Myrtaceae	Tree/Swamp area	Fruit	Mar, Apr, May	Fodder	0.04	[27, 64, 67-69, 70, 77, 78]	MT 031
Tamarindus indica L.	Roqa	Fabaceae	Tree/Swamp area	Fruit	Mar, Apr, May, Sep, Oct, Nov	Medicine (Parasite, parasites, scabies, gastritis, nausea), construction, fuel, fodder	0.14	[28, 59, 65, 68, 70, 82]	MT 022
Vangueria apiculata K.Schum.	Bururi	Rubiacea	Shrub/Forest	Fruit	Mar, Apr, May, Sep, Oct, Nov	Fuel, fodder	0.03	Not reported	MT 008
Ziziphus mucronata Willd.	Kurqura Gebro	Rhamnaceae	Shrub/Forest and Village	Fruit	Mar, Apr, May	Medicine (snake bite), construction, fuel, fodder	0.06	[68]	MT 004
Ziziphus abyssinica Hochst. ex A.Rich.	Kurqura	Rhamnaceae	Shrub/Forest and Village	Fruit	Jun, Jul, Aug	Fence, fuel, fodder	0.04	[64, 68, 70]	MT 020
Ziziphus spina-christi (L.) Desf.	Kurqura Jeneto	Rhamnaceae	Tree/Forest and Village	Fruit	Jun, Jul, Aug	Medicine (dandruff, skin lesion), cosmetics, construction, fuel, fodder	0.6	[64, 65, 70]	MT 003

When comparing wild edible plants and their uses with those of other ethnobotanical studies in Ethiopia, most overlaps of plant resources and their uses were with the study from Hamer and Konso Communities, south Ethiopia (17 species) [68], followed by semiarid Ethiopia (15 taxa) [28], Chilga District, northwestern Ethiopia (10 taxa) [64], Chelia District, west-central Ethiopia and Yilmana Densa and Quarit Districts, Amhara Region (9 taxa each) [27, 69], Yilmana Densa and Quarit Districts, Amhara Region (8 taxa) [67], Berek Natural Forest, Oromia special zone (7 taxa) [4] and Bullen District, northwest Ethiopia (6 taxa) [70] (Table 2). The high overlaps of some plants and their uses with semiarid Ethiopia [28] and Hamer and Konso Communities [68] might be explained by similar cultural structures and geographic similarities (Table 2).

Family Malvaceae was best-represented, accounting for 6 species, followed by Fabaceae and Rhamnaceae (4 species each), Myricaceae (3 species), six families (Apocynaceae, Boraginaceae, Cactaceae, Primulaceae Rubiacea, Salicaceae) consisting of two species, and ten families represented by one species each. The recorded high number of wild edible plants from Malvaceae, Fabaceae and Rhamnaceae might be due to the better adaptation potential of WEPs in these families over wider ranges of altitudes. Likewise, studies performed elsewhere in Ethiopia [64, 65, 71] showed a relatively higher number of wild edible plant families of Malvaceae, Fabaceae and Rhamnaceae. At the genus level, the genera *Acacia*, *Grewia* and *Ziziphus* composed the highest number of species (three species each); they were followed by *Cordia* and *Opuntia* (two species each), and 21 genera included one species each. Similarly, these genera had a higher number of species in a similar ethnobotanical study in Ethiopia, i.e., in the Lower Omo River Valley, the genus *Grewia* constitutes three species [66], in Berek Natural Forest, *Acacia* has two species [4], and in Yalo Woreda, *Ziziphus* contains two species [71].

It should be stressed that one critically endangered and one least concern endemic plant species were found in the IUCN Red List Categories; *Acacia prasinata* Asfaw is a critically endangered species, whereas *Dombeya aethiopica* Gilli is the least concern species [72]. The growth forms (habits) of wild edible plants in Mieso District are shrubs and trees. The dominant growth form (habit) was shrubs (30 species), followed by trees (11 species). There were no wild edible herbs consumed in the study area, which could be because the agro-climatic zone of the study area is semiarid and most of the populations in Mieso practice pastoral and agro-pastoral agricultural systems. Similarly, the wide utilization of shrub growth forms was also reported by Lulekal et al. [22]. However, studies performed in Burji District in Ethiopia [27] and Lhoba people in China [73] reported the dominance of herbs and shrubs.

We calculated the use values (UVs) for each species to determine their relative importance to local people. The six species with the highest use values (UVs) were *F. indica* (Burm.f.) Merr. (1.4), *C. spinarum* L. (1.1), *Z. spina-christi* (L.) Desf. (0.6), *G. villosa* Willd. (0.5), *C. monoica* Roxb. (0.3) and *O. ficus-indica* (L.) Mill. (0.2) (Table 2). The species with the lowest UVs were *Abutilon mauritianum* (Jacq.) Medik., *Acacia tortilis* (Forssk.) Hayne, *D. abyssinica* (A.Rich.) Warb., *Embelia schimperi* Vatke, and *S. americanum* Mill. (0.01 each) (Table 2). It is possible that the higher use value of *F. indica* is related to its multiple uses, such as medicine, construction material, fuel and fodder. *C. spinarum* was also the most preferred species in a study conducted in Ethiopia [64].

### Plant parts used, their habitats and time of gathering

**Fig. 2:**
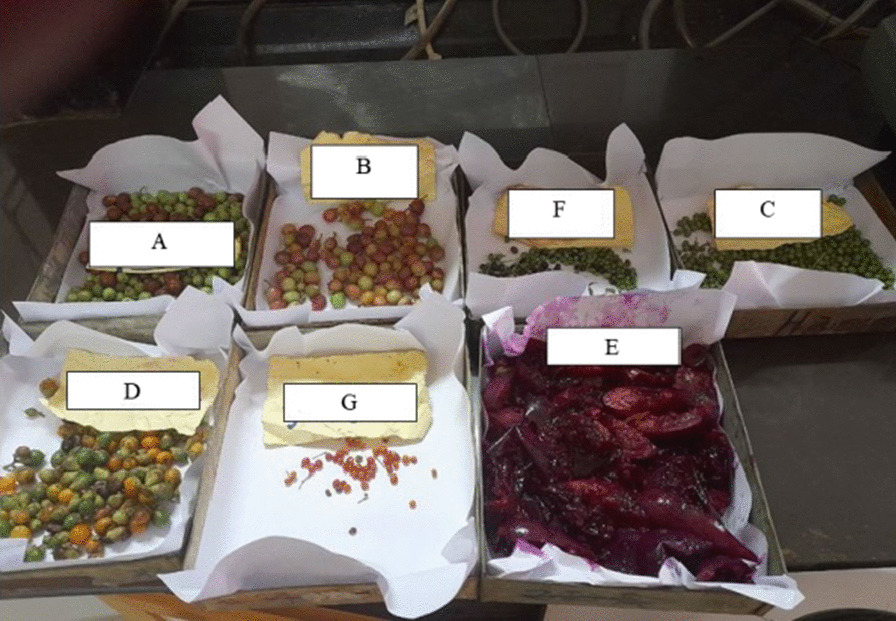
Wild edible fruits in Mieso District (**A**) *Ziziphus spina-christi* (L.) Desf., (**B**) *Vangueria apiculata* K.Schum., (**C**) *Euclea racemosa* L., (**D**) *Cordia monoica* Roxb., (**E**) *Opuntia humifusa* (Raf.) Raf. (**F**) *Flacourtia indica* (Burm.f.) Merr. and (**G**) *Maytenus undata* (Thunb.) Blakelock

The edible plant parts in Mieso District are fruits, roots and bark. The most widely used plant parts were fruits (39 species or 95%), followed by roots and bark (1 species each). This finding is in line with studies performed in Ethiopia [27, 64, 66, 68, 74] and other countries in the world [75, 76] in the sense that fruits are the widely used edible parts. All the recorded edible plants were consumed fresh (see Additional file 1), without additional processing (Fig. 2). The wide use of fruit plant parts is attributable to their day-to-day requirements, ease of processing, nutritional value, and taste [77, 78]. Besides, the agro-climatic zone of the study area is semiarid, and most of the population in Mieso practises pastoral and agro-pastoral agricultural systems. The fruit parts of wild edible plants have high nutritional value, including vitamins, fibres, and secondary metabolites, compared to cultivated crops [79]. In addition, carotenoids, copper-rich mesocarps, proteins, and minerals such as magnesium, phosphorus and copper are also obtained from wild edible fruits [79]. In Ethiopia, the crude protein, crude fibre, moisture content, carbohydrate, total energy and mineral contents in the fruits of *Balanites aegyptiaca* (L.) Delile, *Cordia africana* Lam. and *Z. spina-christi* (L.) Desf. were determined. The fibre contents of *B. aegyptiaca* (L.) Delile and *Z. spina-christi* (L.) Desf. were higher, while carbohydrate and energy contents were found to be higher in the fruits of *Z. spina-christi* (L.) Desf. A higher value of calcium in the fruits of *B. aegyptiaca* (L.) Delile and *Z. spina-christi* (L.) Desf. were found, whereas iron, zinc and potassium contents in the fruits of *B. aegyptiaca* (L.) and *C. africana* Lam were higher than the value found in *Z. spina-christi* (L.) Desf. by approximately 50% [80].

**Fig. 3:**
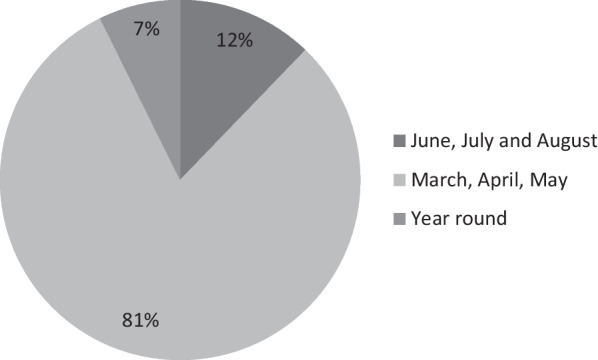
Months of collection for WEPs

The plant parts used in Mieso were gathered in different seasons/times for collection. Most WEPs are collected from March to May (*Badheysa*) (33 species), gradually decreasing from *Ganna* (June, July, August; 5 species). Only three species were collected year round (Fig. 3). *Badheysa* periods are marked with the beginning of slight rain. The main species collected in this season include *C. spinarum* L., *Vangueria apiculata* K. Schum., *Berchemia discolor* (klotzsch) Hemsl., *Acokanthera schimperi* (A.DC.) Schweinf., *G. villosa* Willd. and *Dovyalis abyssinica* (A.Rich.) Warb. The main plants collected in the *Ganna* season (from June to August) include *A. prasinata* Asfaw, *Hibiscus micranthus* L.f., *Solanum americanum* Mill., *Z. spina-christi* (L.) Desf. and *Hydnora abyssinica* A.Br. The high uses of wild edible plants from March to May (*Badheysa;* rainy season) might be due to the high resprouting time for most WEPs. Besides, this season is the flowering time for most WEPs, and their fruiting is also harvested during this season. During the dry season, rural people rely heavily on a stored diet [76]. The relative seasonal availability of WEPs highly affects the nutritional and food insecurity of households [76]. Wild edible plants supplement the human diet by adding various vitamins, flavours and minerals during food scarcity [81].

The people in Mieso District collect wild edible plants from various habitats, including forest habitats, villages and swamp habitats. The largest number (23 species) was collected from forest habitats only, followed by both village and forest habitats (8 species), swamp habitats (6 species) and village habitat only (4 species) (Table 2). Ethnobotanical studies on WEPs in different parts of Ethiopia [27, 28, 77] showed that most WEPs were gathered from forest habitats.

The majority of wild edible plants (28 species or 68%) were consumed only during famine or in times of food shortages followed by supplementing staple foods (9 species or 22%) and only for their taste (4 species). The main famine plants are *Plectranthus montanus* Benth., *V. apiculata* K.Schum., *Rhus natalensis* Bernh. Ex C.Krauss, *Pappea capensis* Eckl. & Zeyh., *Maytenus undata* (Thunb.) Blakelock, *A. prasinata* Asfaw, *C. monoica* Roxb., *Euclea racemosa* L., *Mimusops kummel* Bruce ex A.DC., *Z. spina-christi* (L.) Desf., *Myrsine africana* L., *H. micranthus* L.f. and *S. americanum* Mill. Famine WEPs play an important role in the survival of rural communities during periods of food scarcity [84]. However, some WEPs cause harmful effects when consumed in large quantities. For example, informants mentioned that *O. ficus-indica* (L.) Mill. causes diarrhoea and bloating, *C. spinarum* L. and *Balanites aegyptiaca* (L.) Del. causes abdominal pain when consumed in larger quantities.

### Multiple uses of wild edible plants

The people in Mieso also use wild edible plant resources for other multiple uses, including for fodder, fuel, medicine, construction, cosmetics and bee keeping (Table 3). Twenty-three species were mentioned for fodder use, followed by fuel purpose (21 species), medicinal value (13 species), construction (10 species), cosmetics (4 species), bee keeping (2 species), and others, such as detergent substitutes (for head, hand and clothes), fencing, cleaning teeth and shade (1 species each) (Table 3). The most frequently mentioned species for fodder use were *C. spinarum* L., *F. indica* (Burm.f.) Merr., *G. villosa* Willd., *Z. spina-christi* (L.) Desf., *B. discolor* (klotzsch) Hemsl., *R. natalensis* Bernh. ex C.Krauss., *P. capensis* Eckl. & Zeyh. and *C. monoica* Roxb (Table 2). Informants mentioned that *R. natalensis* Bernh. ex C.Krauss is good fodder for cattle and camels. *C. spinarum* L. and *P. capensis* Eckl. & Zeyh. are used for bee keeping practices. *G. villosa* Willd. was used as a detergent substitute (for head, hand and clothes), and *C. spinarum* L. was also used for cleaning teeth.

**Table 3:** Multipurpose plant species

Category	No. of species	%
Fodder	23	30
Fuel	21	27
Medicine	13	17
Construction	10	13
Cosmetic	4	5
Bee keeping	2	3
Detergent substitutes	1	2
Fencing	1	1
Teeth brush	1	1
Shade	1	1

In addition, wild edible plants in Mieso District are also used in medicine, and these dietary medicines are called nutraceutical plants/dietary medicines [41, 78]. Livestock ailments such as retained placenta, eye diseases, and diarrhoea and human ailments such as cancer, wounds, malarias, hepatitis, bleedings, dandruff, headache, bone fracture, anemia, nausea, scabies, intestinal parasites, and bloating are reported to be treated by nutraceutical/medicinal dietary plants in Mieso District (Table 2). Used plant parts of medicinal dietary plants for traditional medicine are roots, leaves, fruits and bulbs. Parts used for three medicinal dietary plants were roots, fruits and bulbs; leaves were the other used plant parts of ten species. The fruit of *Tamarindus indica* L. was used to treat intestinal parasites, scabies, gastritis and nausea, and the leaves of *Z. spina-christi* (L.) Desf. against dandruff and skin disease, *Ziziphus mucronata* Willd. against snake bites, the leaves of *C. monoica* Roxb. against itching, *M. africana* L. against intestinal parasites, *B. aegyptiaca* (L.) Delile against bloating, and five species, i.e., *F. indica* (Burm.f.) Merr., *Grewia ferruginea* Hochst. ex A.Rich., *H. abyssinica*, *Breonadia salicina* (Vahl) Hepper & J.R.I.Wood, *R. natalensis* Bernh. ex C.Krauss were used against cancer (Table 2). For livestock ailments, the roots of *E. racemosa* L. is crushed and applied on the infected eyes of livestock, and the juice is taken orally for treating diarrhoea (Table 2). Nutraceutical plants are used to address a wide range of health problems and as a source of foods [85]. The medicinal and dietary uses of nutraceutical plants such as *B. aegyptiaca* (L.) Delile, *R. natalensis* Bernh. ex C.Krauss, *T. indica* L., *Z. spina-christi* (L.) Desf. and *M. africana* L. have also been reported in studies conducted elsewhere in Ethiopia [19, 71, 83].

Four species in the study area were used in traditional cosmetic applications*.* The bark of *G. villosa* Willd, the leaves of *Z. spina-christi* (L.) Desf. and *C. spinarum* L. were used for beautifying and softening hair. *F. indica* (Burm.f.) Merr. was used for whitening and improving females’ faces. The crushed leaves of *G. villosa* Willd were used as soap for washing the hair. *C. spinarum* L. and *P. capensis* Eckl. & Zeyh. were used for bee keeping practices (Table 2). Nevertheless, the utilization of WEPs for multiple uses in conjunction with overharvesting for edibility results in losses of plant resources [6]. Thus, the protection and sustainable use of these plants must be valued and considered. Currently, due to living standard improvements in different communities, the need for food balance and food varieties is increasing; hence, edible plant resources in the wild are important for domestication and variety selection [86].

The most commonly used WEPs in terms of citations were *C. spinarum* L. (45 citations), *F. indica* (Burm.f.) Merr. (43 citations), *B. discolor* (klotzsch) Hemsl.*, G. villosa* Willd. and *O. ficus-indica* (L.) Mill. (20 citations each), *Z. spina-christi* (L.) Desf. (17 citations), *C. monoica* Roxb. (11 citations) and *T. indica* L. (6 citations) (Table 4). *Z. spina*-*christi* (L.) Desf.*, T. indica* L. and *B. aegyptiaca* L. were also among the highly cited species in Quara District northwest Ethiopia [65].

**Table 4:** Most frequently cited WEPs

Species	Citation
*Carissa spinarum* L.	45
*Flacourtia indica* (Burm.f.) Merr.	43
*Berchemia discolor* (klotzsch) Hemsl.	20
*Grewia villosa* Willd.	20
*Opuntia ficus-indica* (L.) Mill.	20
*Ziziphus spina-christi* (L.) Desf.	17
*Cordia monoica* Roxb.	11
*Tamarindus indica* L.	6

### Commercial valuation

In addition to daily material supply, WEPs play a vital role in ethnic groups’ cash income. There are 11 wild edible plants sold at the local market in Mieso market centres (Table 5). The most important was *F. indica* (Burm.f.) Merr. mentioned by 20 informants, followed by *Z. spina-christi* (L.) Desf. (14 mentions), *T. indica* L. (6), *C. spinarum* L. (5), *B. discolor* (klotzsch) Hemsl. (4), *O. ficus-indica* (L.) Mill. (3) and *G. villosa* Willd. (2) (Table 5). For example, the fruit of *F. indica* (Burm.f.) Merr. was sold for 50 Birr/kg, *Z. spina-christi* (L.) Desf. 30 Birr/kg, *T. indica* L. 20 Birr/kg, *C. spinarum* L. 40 Birr/kg, *B. discolor* (klotzsch) Hemsl. 25 Birr/kg and *O. ficus-indica* (L.) Mill. 25 Birr/kg. In contrast, because of their ample supply and wide distribution, the price of *G. villosa* Willd., *H. abyssinica* A.Br., *R. natalensis* Bernh. ex C.Krauss, *M. kummel* Bruce ex., and *M. africana* L. was much lower (10 Birr/kg each) (Table 5). Species such as *T. indica* L., *Z. spina-christi*, *C. spinarum* L., *H. abyssinica* A.Br. were sold for medicinal value and edibility, whereas the rest were sold for edible purposes. Similarly, the fruits of species such as *T. indica* L., *C. spinarum* L., and *M. kummel* were marketable wild edible plants in studies conducted elsewhere in Ethiopia [64, 77, 85]. Such plants can provide hints about flora resources that have commercial value associated with the knowledge of local people. Besides, safe economic exploitation is important for improving neglected wild food plant utilization and for promoting development [54].

**Table 5:** WEPs sold in Mieso

Plant species	Cost	Number of mention
*Flacourtia indica* (Burm.f.) Merr.	50 birr/kg	20
*Ziziphus spina-christi* (L.) Desf.	30/kg	14
*Tamarindus indica* L.	20 birr/kg	6
*Carissa spinarum* L.	40 birr/kg	5
*Berchemia discolor* (klotzsch) Hemsl.	25/kg	4
*Opuntia ficus-indica* (L.) Mill.	25 birr/kg	3
*Grewia villosa* Willd.	10 birr/kg	2
*Hydnora abyssinica* A.Br.	10 birr/kg	1
*Rhus natalensis* Bernh. ex C.Krauss	10 birr/kg	1
*Mimusops kummel* Bruce ex	10 birr/kg	1
*Myrsine africana* L.	10 birr/kg	1

### Threats to wild edible plant knowledge and use

**Fig. 4:**
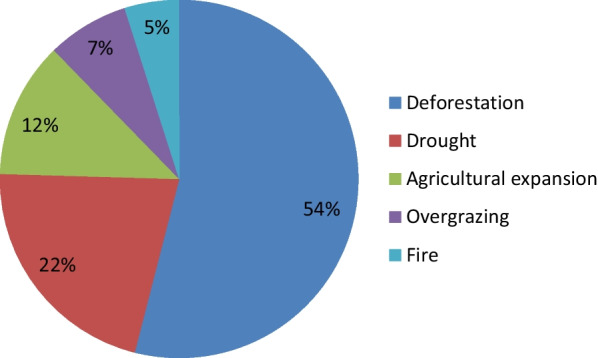
Threats to wild edible plants in the study area

According to informants from all villages, several factors threatened wild edible plants in Mieso District. The main threat to wild edible plant resources mentioned by the respondents was deforestation, with 88 mentions (54%), followed by drought (35 mentions or 22%) and agricultural expansion (20 mentions or 12%) (Fig. 4). Similarly, these threats to WEP resources have been reported in ethnobotanical studies conducted in Ethiopia [4, 22, 64, 65, 77, 87, 88]. Semistructured interviews and focus group discussions with informants showed that plant species, such as *T. indica* L., *D. abyssinica* (A.Rich.) Warb., *G. villosa* Willd., *H. abyssinica* A.Br., *M. kummel* Bruce ex A.DC., *Cordia africana* Lam., *M. africana* L., *P. montanus* Benth., *A. schimperi* (A.DC.) Schweinf., and *R. natalensis* Bernh. ex C. Krauss were rarely encountered.

Most participants (57 or 85%) gained their wild edible plant knowledge from parents, followed by elders (8 or 12%) and friends and relatives (2 or 3%). All of the respondents transfer their knowledge of WEPs orally along the family line. Most of the informants, 27 (66%), were willing to transfer their knowledge to their children, followed by 8 (19.5%) for friends and 6 (14.6%) for any person. Similarly, studies from different countries have reported that sociocultural influence was the main factor for the neglected consumption of WEPs [89, 90].

Land-use changes and direct exploitation of plant resources also explain the decreased abundance of WEPs [91–93]. In addition, habitat destruction and overharvesting also pose significant impacts on wild plant resources [6]. In addition to plant resources, traditional knowledge associated with this resource is also gradually disappearing. The overuse of some plants for several uses (fodder, medicinal, edible, etc.) was also a factor in the rarity of some wild edible plants in the study area. According to informants, more than one method of protection was recommended. Accordingly, awareness creation was recommended most to protect and conserve wild edible plants, 50 mentions (29%), followed by on-site conservation (34 mentions or 20%), soil and water conservation (29 mentions or 17%), home garden conservation (27 mentions or 15%), fencing (25 mentions or 14%) and reforestation (8 mentions or 5%). Cultivating wild edible plants in home gardens and conserving and protecting them in the wild/natural setting (in situ) is important to guarantee future access to wild edible plants for dietary supplements and for the healthcare system of local people and for laboratory investigations to obtain new chemical-lead findings.

## Conclusions

From this study, traditional knowledge on wild edible plant use has been revealed, and it is still practised by the ethnic community of Mieso District. Informants rely on wild edible plant resources for dietary supplementation, during famine, and for income generation. The cultural uses of the plants in the study area also overlap with the use in other parts of Ethiopia, indicating some knowledge and use similarities with the respective areas. For ethnic identities and culture conservation, subsistence with conventional food is an important tool. Hence, the contribution of rural communities to the diversification of nutrition should be recognised and acknowledged for ethnic knowledge reappraisal. Wild fruits and vegetables with market potential can be income sources for residents. WEPs that have excellent traits can be protected and preserved by cross-breeding new plant varieties. More comprehensive analysis of the mineral composition, nutritional value and biological activity of WEPs is being performed. Therefore, this study makes a significant contribution to the preservation of wild edible plants and uses in this district.

## Supplementary Information

**Additional file 1. **Photograph illustrating wild edible fruits in Mieso District.

## Data Availability

All data generated or analysed during this study are included in this manuscript.

## Abbreviations

ANOVA: Analysis of variance
CSA: Central statistical agency
HH: Household
UVs: Use values
WEPs: Wild edible plants
