# Supplementary material for: The traditional use of wild edible plants in pastoral and agro-pastoral communities of Mieso District, eastern Ethiopia
Source: Trop Med Health. 2023 Feb 23;51:10. doi: 10.1186/s41182-023-00505-z (PMC9948460; doi:10.1186/s41182-023-00505-z)
Supplement: Supplementary file 1 — Additional file 1. Photograph illustrating wild edible fruits in Mieso District. [file 41182_2023_505_MOESM1_ESM.docx]

Additional file 1. photograph illustrating wild edible fruits in Mieso District

*
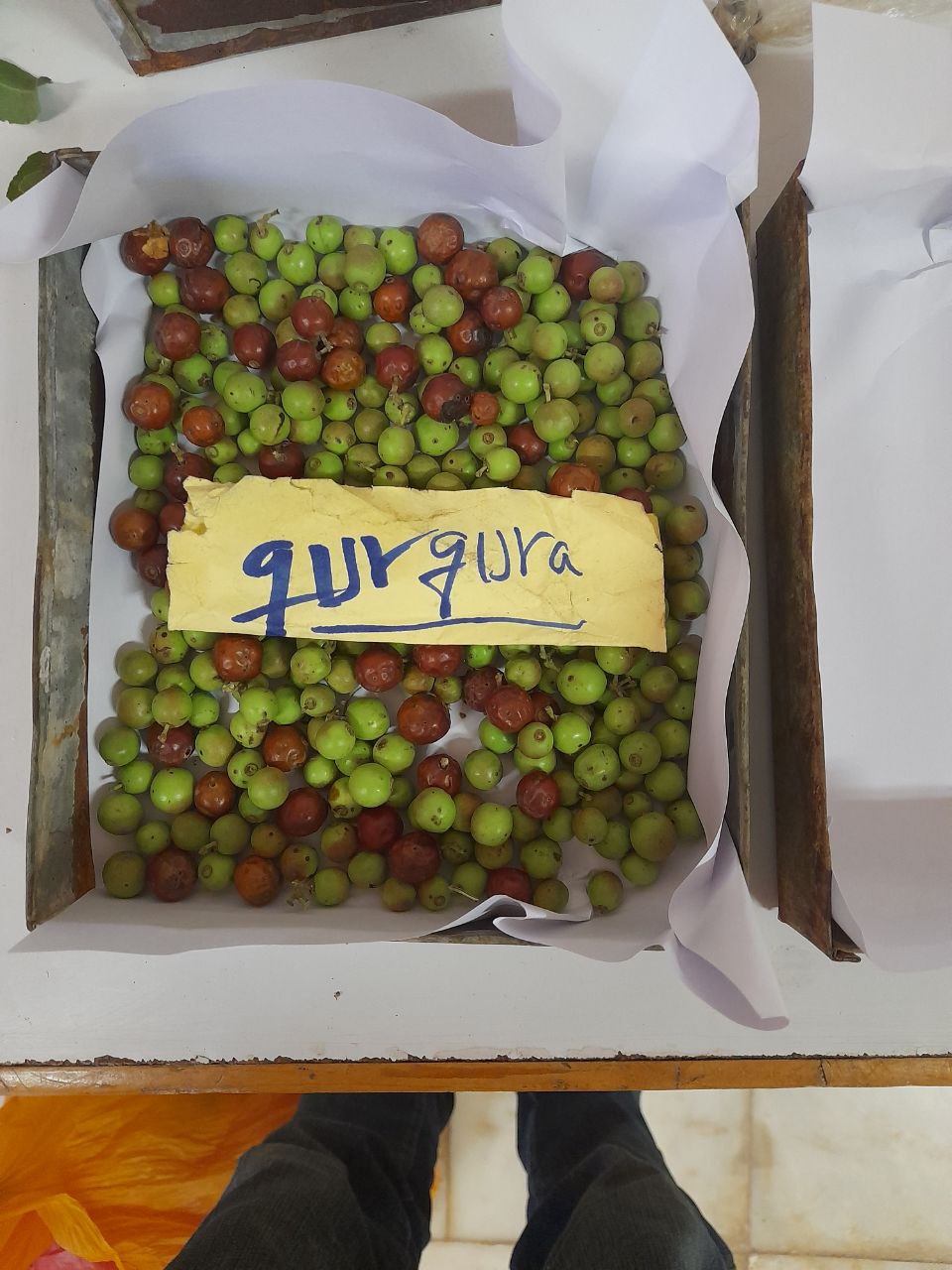
*

**Figure S1** *Ziziphus spina-christi* (L.) Desf. (Photo credit: Muhidin Tahir)

*
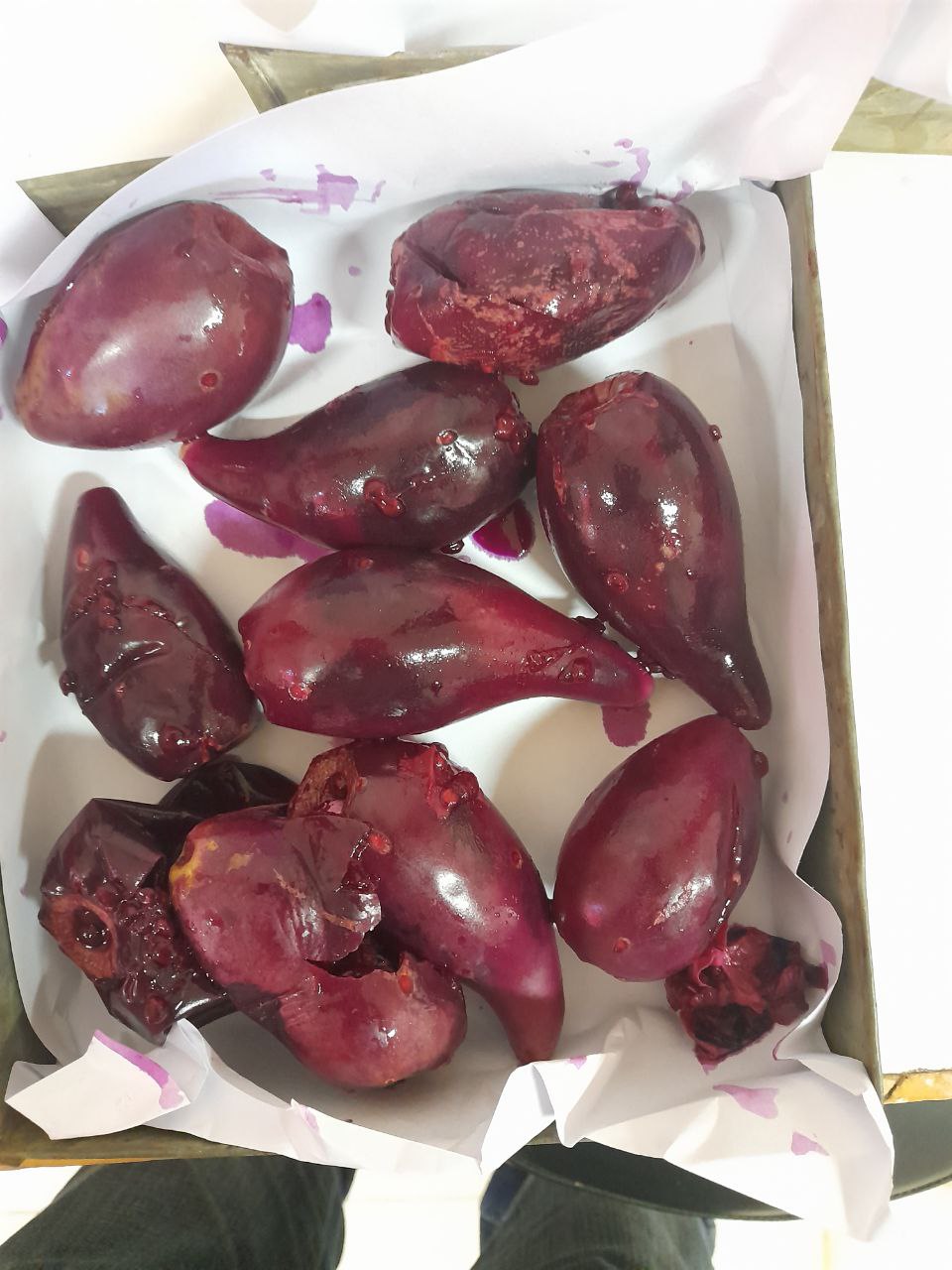
*

Figure S2 *Opuntia humifusa* (Raf.) Raf. (Photo credit: Muhidin Tahir)

*
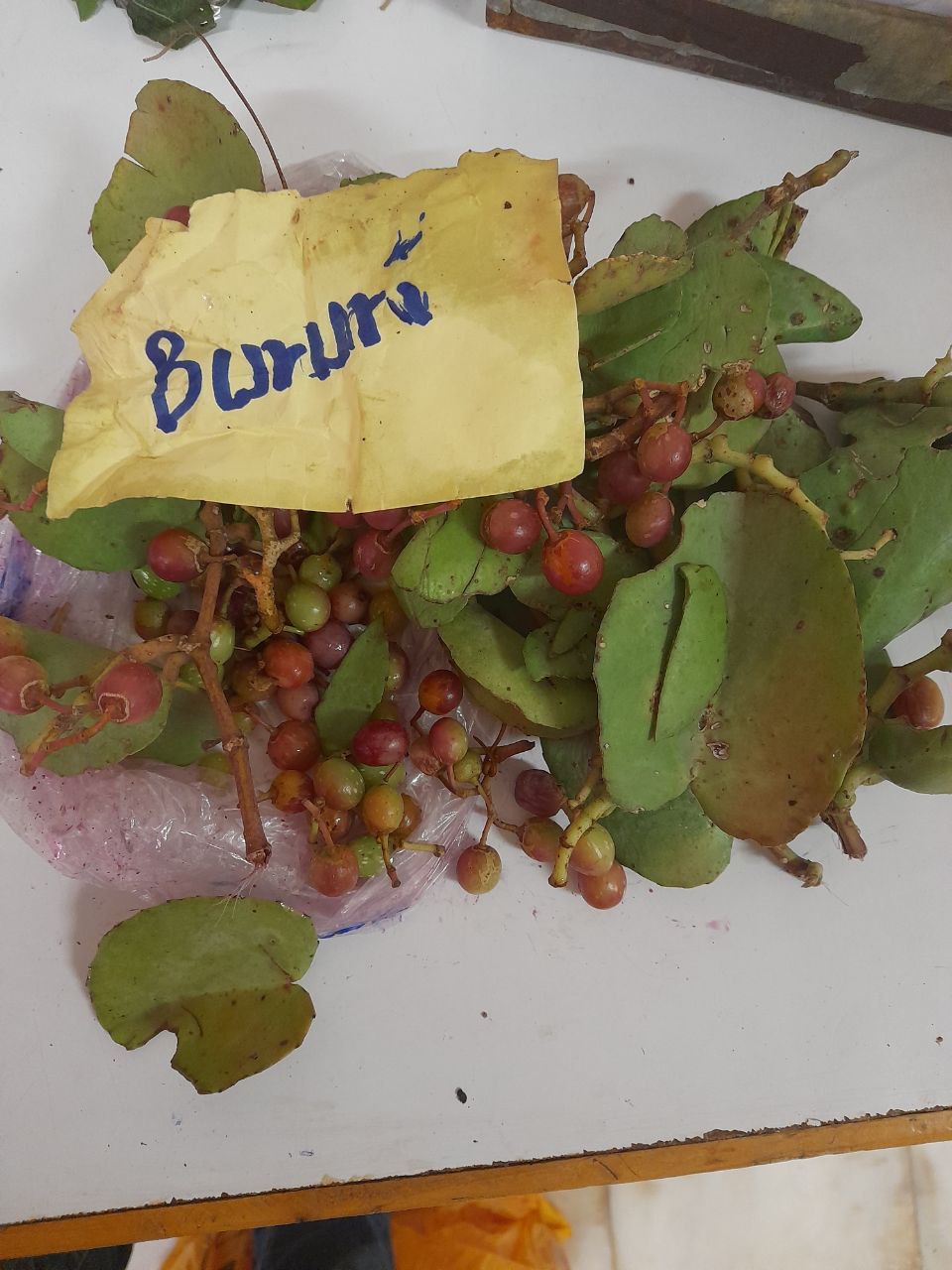
*

Figure S3: *Vangueria apiculata* K.Schum. (Photo credit: Muhidin Tahir)

*
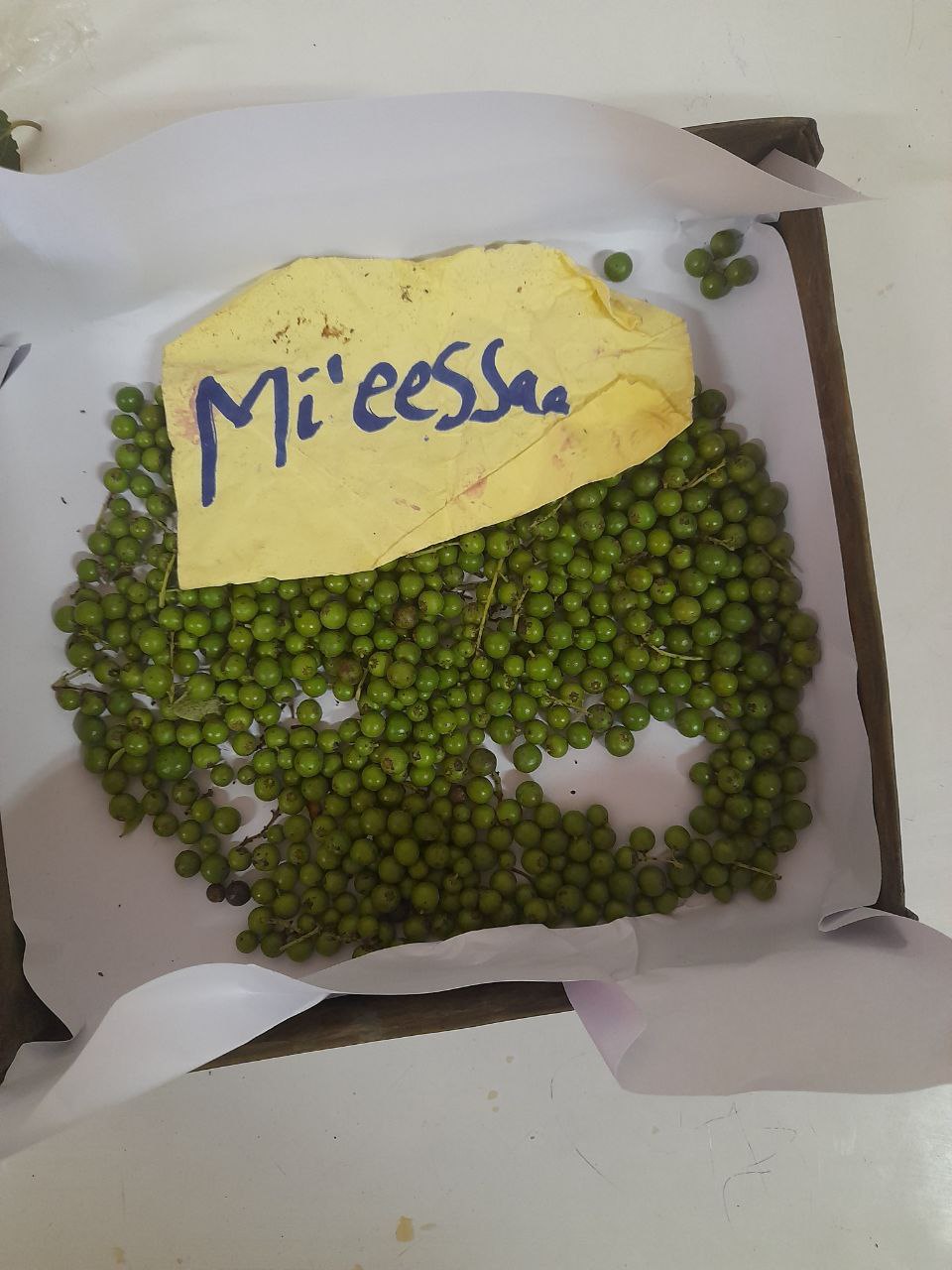
*

Figure S4: *Euclea racemosa* L. (Photo credit: Muhidin Tahir)

*
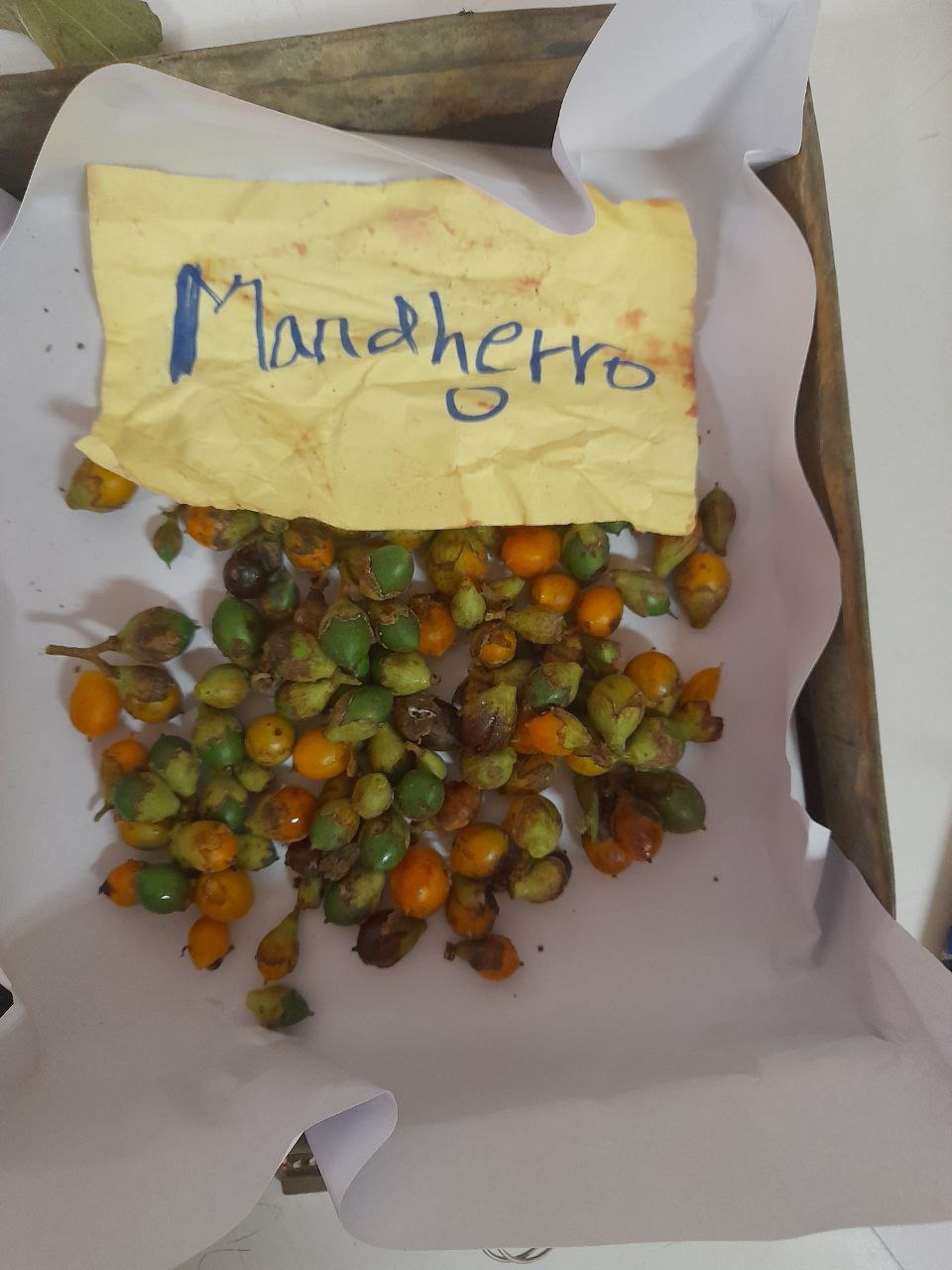
*

Figure S5: *Cordia monoica* Roxb. (Photo credit: Muhidin Tahir)
